# Epigenetic drugs: from chemistry via biology to medicine and back

**DOI:** 10.1186/s13148-016-0222-5

**Published:** 2016-05-23

**Authors:** Lucia Altucci, Marianne G. Rots

**Affiliations:** Seconda Università degli Studi di Napoli, Dipartimento di Biochimica, Biofisica e Patologia generale, Vico L. De Crecchio 7, Napoli, 80138 Italy; University Medical Center Groningen, Groningen, Netherlands

The so-called epigenetic drugs are a relatively new class of drugs acting on chromatin enzymatic and non-purely enzymatic complexes, for which interest has grown exponentially in the last decade. Indeed, huge research efforts have been initiated within academia to address chemical as well as biological aspects of epigenetic drugs and their targets. This growing interest is paralleled by strong investments in exploring new epigenetic drugs by (big) pharma. In addition, the interest of clinicians to explore epigenetic mechanisms and epi-marks as therapeutic tools for a patient’s stratification and therapy is currently reaching far beyond the initial field of oncology.

Despite the growing awareness of the role of epigenetic dysregulation as cause-and/or-effect in disease and the exploding number of modulating compounds, many aspects remain unaddressed and require further improvement.

Given this urgent need, we present in this series an overview of state-of-the-art knowledge of drug design for old and new epi-targets, their mechanisms of actions, and the increasing spectrum of clinical applications. In addition, we introduce an European Cooperation in Science and Technology (COST) platform to address open questions, directions, and potential emerging concerns (see Table 1).

**Table 1:** Content of Review Series "Epi-Drugs: from chemistry via biology to medicine and back"

**Content of Special Issue Clinical Epigenetics:**
**Epigenetic drugs- from chemistry via biology to medicine and back**
**I. General introduction to epigenetic drugs and ongoing actions**
1. A short general statement to introduce this series of reviews
2. An overview of EPICHEMBIO COST action
**II. Old targets, new drugs**
3. DNMT inhibitors
4. HDAC inhibitors
5. SirT modulators
**III. Novel epigenetic targets and new drugs**
6. HAT inhibitors
7. BRD hitting drugs
8. Histone methyltransferases and demethylases
9. EZH2
10. LSD
11. Multi-Target epidrugs
**IV. Status of animal studies, potential clinical applications/current trials**
12. Oncology, solid cancers Prostate
13. Onco-Hematological
14. The case of MM
15. Metabolic dysregulations
16. Neurodegenerations/ageing
17. Regenerative Medicine
**VI. Where to take it from here: (off)target identification and validation approaches**
18. Studying epigenetic complexes and their inhibitors with the proteomics toolbox
19. Epigenetic assays for chemical biology and drug discovery
20. New technologies basic/clinical epigenomics

One of the concerns is the relatively wide effect of epigenetic modulating mechanisms: next to the intended upregulation of, for example, tumor suppressor genes in cancer, metastasis genes might also become upregulated as an unwanted side effect. This realization results in seemingly contradictory approaches of exploring synergisms of inhibiting writers (DNA methyltransferase (DNMTs)), while increasing the substrate of these enzymes (SAM) to stimulate activity [1]. Similarly, efforts are being undertaken not only to inhibit HDAC activity as an anticancer approach but to focus on identifying HAT inhibitors as well. The underlying rationale of such counterintuitively contradictory approaches is based on differential gene expression dysregulation associated within any given diseases: some genes are aberrantly overexpressed while others are repressed.

The success of such opposing strategies can also be explained by our current lack of knowledge on the mechanism of action of the enzymes: largely unknown parameters are involved including complex formation, microenvironment conditions [2], and context-specific control. Upcoming technologies such as multi-omics single-cell analyses [3] and epigenetic editing [4, 5] may allow localized detections and gene-targeted changes, which might turn essential in fine-tuning such parameters. Eventually such insights may also allow a better rational design of drugs (and combination of), further exploiting the promise of epigenetic drugs and potentially the revitalization of the so-called old drugs.

In addition to the current lack of knowledge on mechanisms of action on chromatin modulation and dedicated epidrugs, we have only slowly started to unravel the wide spectrum of substrates of the supposed epigenetic enzymes. Epidrugs are designed to inhibit (or activate) histone-modifying enzymes or DNA methyltransferases, or to interfere with readers of the resulting chromatin modifications. However, it is more and more accepted that these chromatin modifiers (and the dedicated epidrugs) affect various other classes of substrates, including proteins in signaling pathways and cellular architecture. If, for oncology applications, this might be an additional advantage as cancer cell death is the intended ultimate outcome, for other clinical applications, greater insight into the long-term effects is warranted.

Learning from the present knowledge, valproic acid has been prescribed for epilepsia patients for several decades now, without reporting serious side effects [6], and even some cancer-preventive effects were documented [7]. Comparably, 5aza, first tested on Myeloid Dysplastic Syndrome patients, did not display important side effects even when a long-term vision was applied, suggesting that worries might rely more in acquired resistances than in intrinsic toxicity.

These two drugs are mere examples of the principal two classes of epidrugs to obtain FDA approval: DNA methyltransferase inhibitors for treatment of MDS (azacitidine, in 2004) and histone deacetyltransferase inhibitors for T cell lymphoma (Vorinostat, in 2006). Currently, five histone deacetylase inhibitors and two DNA methyltransferase inhibitors are FDA approved, and developments in their respective areas are discussed in reviews #3, #4, and #5.

As a results of the growing awareness of the wide spectrum of epigenetic mutations and deregulations underlying cancer (and many other diseases), more and more insights are obtained on the (dys)function of other chromatin modifying enzymes, and many of these are currently explored as therapeutic targets as well as potential bio-markers (series review #s 6–11).

Although all new therapeutic targets face challenges when tested in biological systems, for epigenetic enzymes this is even more complex: How to interpret cell toxicity assay data for inhibitors of histone-modifying enzymes which actually do affect many more pathways in the cellular compartment? What degree of differential effects can be attributed given that the effect of the enzymes is frequently dependent on the complex the enzyme functions in?

The final outcome of a novel drug may not rely on the catalytic action or at least not only. In addition, 3D chromatin structure and the interplay of stable and transitory interactions (and potential priorities dictated by the presence or the absence of some deposited marks) should be also taken into account suggesting a further level of complexity for chromatin complexes (and epi-enzymes). Finally, the functional effect of epigenetic modifications with respect to gene expression is highly context-dependent.

Altogether, inhibitors (and the few activators known so far) of epigenetic enzymes should be tested in carefully chosen biological systems, incorporating adequate readout assays and solid controls. Series reviews #s 12–16 set out to address such biological questions and provide an overview of the status of several epigenetic drugs in oncology, metabolic dysregulation, and neurodegeneration. Finally, the promises of epigenetic drugs in regenerative medicine are also discussed (review # 17).

The last part of the series (series review #s 18–20) addresses important technological advances to be taken into consideration for the development of potent epigenetic drugs. Overall, we feel that the current “epi-drug era” opens novel avenues for various diseases beyond cancer, but several aspects should be taken into account to fulfill the promise of reversing epigenetic mutations by genome-wide acting epigenetic enzyme inhibitors.
